# Perceptions of Environmental and Health Effects of Quarry Activities at Klefe in the Ho Municipality of the Volta Region

**DOI:** 10.1029/2024GH001168

**Published:** 2025-03-06

**Authors:** Selase Kofi Adanu, Maxwell Kwame Boakye, Shine Francis Gbedemah, Mexoese Nyatuame

**Affiliations:** ^1^ Department of Environmental Science Ho Technical University Ho Ghana; ^2^ Department of Geography and Earth Sciences University of Environment and Sustainable Development Somanya Ghana; ^3^ Department of Agricultural Engineering Ho Technical University Ho Ghana

**Keywords:** quarry, environmental, health effects, livelihoods, degradation, pollution

## Abstract

Expansion of residential and commercial facilities have contributed to rapid urbanization of Ho municipality. As a result, quarry activities have intensified in Klefe a major stone quarry source for construction. The increased demand for quarry stones has created jobs for many but has also led to the perception of health and environment challenges negatively affecting the people and the natural environment. In view of the extensive quarry activities in the area, the aim of the study was to assess perceptions of effects of quarrying activities on human health and the environment. Stratified random sampling method was used to select respondents to answer questions on a questionnaire and application of a relative importance index to examine what the study community perceive as the most important environmental and health effects of quarrying. Exploratory factor analysis was used to determine relationships existing among environmental hazards and their perceived health effects. Sedimentation, land degradation and injury from quarrying were the main perceived effects of quarrying. Efforts to address any perceived effects of quarrying should focus on sedimentation, land degradation and injury from quarrying.

## Key Points

- Streams and rivers are perceived to have sediments as a result of quarrying
- Vegetation and soil degradation are perceived to have negatively affected the livelihood of the people
- Perceived health effects of quarrying include injuries and respiratory diseases

## 1 Introduction

Quarrying which refers to the extraction of rock resources on the surface or below the earth has created job opportunities for many and also contributed to some environmental and health challenges (Banez et al., 2010). Expansions in construction industries have accelerated the global demand for quarry stones which was 5.73 billion US dollars in 2023 and projected to reach 7.75 billion US dollars by 2032 (Business Research Insights, 2024). In the Ho Chi Minh megapolis of Vietnam, the increasing demand for quarry stones has increased the quantity of stones mined to 181 million m^3^ in 2020 (Bui et al., 2020). For the best production outputs to be achieved, and for minimal environmental damage to occur, the choice of quarry sites is often influenced by quarry locations and layouts as these factors influence the choice of tools needed to work on the quarry sites (Botka et al., 2019). Unplanned and unscientific quarrying methods in the Shimla District of India for example, have been responsible for unstable slopes, recurring creeps, landslides, vegetation depletion, health and environmental problems (Kumar, 2020).

Environmental and health concerns are often associated with quarry sites in Africa. In Border II sub‐location in Kenya, quarrying activities are perceived to have problems of dust, noise pollution, land degradation, vegetation loss and vibrations (Opondo et al., 2022). Furthermore, socio‐economic impacts are cracks in buildings, injuries, and road damages (Opondo et al., 2022). It is also worth noting that it is not in all situations that destructions at quarry sites are attributed to quarry activities alone. In South Sudan for example, studies have shown that the vegetation cover loss at quarry sites was not solely as a result of quarrying activities but other factors such as sand winning (Moilinga & Athian, 2022).

Quarrying activities are sources of dust pollution on agricultural lands, soil erosion and soil productivity loss (Ali et al., 2020). Other effects of quarrying are the production of waste, damage to biodiversity, habitat loss and fragmentation (Anand, 2006; Wang, 2007). Besides the environmental effects of quarrying, health effects such as body pains, eye infections, sleepless nights, headaches, coughs and chest pains are effects associated with quarrying (Mahapatra, 2023). The health impact of quarrying activities are potentially linked to air borne dusts. The air borne dust particles are usually very minute, thus particles that are less than 10 microns (<10 µm) in diameter. This particulate matter affects the respiratory and cardiovascular systems of the body if inhaled. The impact of the dust effect in the local community can be up to a 1 km radius from the epicenter of the quarry activity, however, the worse effects are usually within 100 m (Okafor et al., 2023).

The hydrological cycle is affected when the vegetation cover (flora) is removed at quarry sites leading to reduction in stream flows and a slowdown in ground water recharge (Saxena et al., 2021). Quarrying affects water quality as well as quantity. Laboratory analysis of water samples from quarry pits and nearby shallow wells have shown differences in pollution levels as dust emission was found to be higher than the recommended permissible standards. (Bakamwesiga et al., 2022).

Quarrying can also result in the loss of natural carbon sinks. Thus, quarrying influences climate change and variability, increase carbon footprint and hence the need to adopt smart technology for sustainable quarrying (Ruiz et al., 2023).

In the Ashanti region of Ghana, negative effects of quarrying activities reported at Buoho quarry site were respiratory diseases, eye problems, muscle pains and malaria. Also, dust emission was reported as result of crushing of stones and trucks conveying stones from the quarry site (Asante et al., 2014). Quarrying in Daglama in the Ho Municipality of the Volta Region has contributed to the destruction of farm lands and forests (Bewiadzi et al., 2018).

The study objectives were to find out perceived health challenges associated with quarrying and the perceived consequences of quarrying on land, plants and water bodies. Even though the study did not measure effects of stone quarrying in the study community, views of those engaged in quarrying activities reflect their beliefs that quarrying is beneficial but also has negative effect on their health and the environment as such there is a need for caution while working at the quarry sites.

## 2 Materials and Methods

### 2.1 Study Area

**Figure 1:**
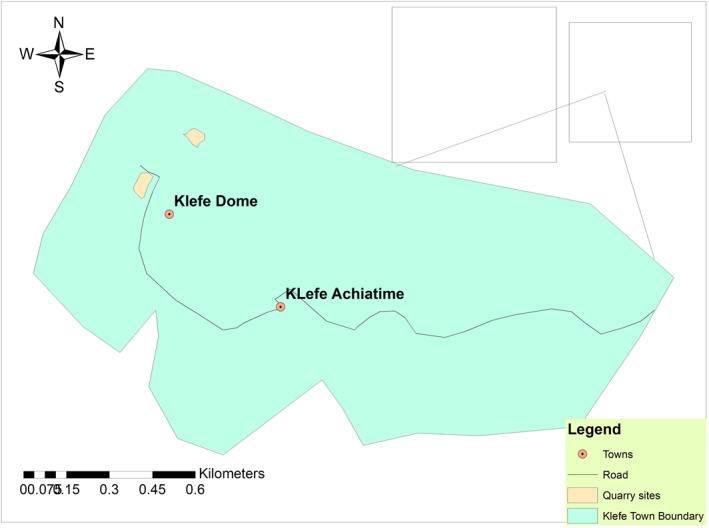
Map of Klefe.

The Ho Municipality is located between latitudes 6°20″N and 6°55″N and longitudes 0°12′E and 0° 53′E and covers a total land area of 587 km^2^ and has a human population of 180,420 (Ghana statistical Service, 2021). The municipality shares boundaries with Adaklu and Agotime‐Ziope Districts to the South, Ho West District to the North and West, and the Republic of Togo to the East. The municipality includes a mix of mountainous and lowland areas and the Klefe stone quarry site is located on the north eastern part of the mountain range which is covered by moist semi‐deciduous forest and woodlands (Figure 1).

### 2.2 Determination of Sample Size

The sample size was estimated using a formula developed by Yamane (1967). It was calculated as:

$$n=\frac{N}{1+N{\left(e\right)}^{2}}$$

Where *n* is the sample size, *N* is the population size of quarry workers and individuals living around the quarry site, and *e* is the level of precision. Using a confidence level of 95%, a level of precision of 5% (0.05), and a population size (*N*) of 426, the sample size (*n*) of 206 was obtained. Due to the corporation with the quarry workers in participating in this study, the sample size was increased to 225 respondents.

### 2.3 Sampling Procedure and Data Collection

Respondents were chosen using simple random sampling. The population of the study comprised quarry workers, individuals living in the vicinity of quarry site, and a site manager. The purpose of the research was made known to the local community, opinion leaders and the quarry workers as such their consent was sought before undertaking the research. Ethical approval for the study was obtained from the Ho Technical University Ethics Committee with reference number HTU/DRI/EC/VOL.I/24/023. Face to face interviews were conducted to collect data from the respondents by asking questions using questionnaire developed in English but interpreted in the local Ewe language.

### 2.4 Instrument Used for Data Collection

The questions were adapted after a meticulous review of the relevant literature on environmental and health effects of quarry activities to identify the appropriate issues to ask about. The instrument consisted of a structured questionnaire based on related studies (Bewiadzi et al., 2018; Bui et al., 2020; Ghosh & Lohe, 2022; Mahapatra, 2023; Opondo et al., 2022). A total of 14 indicators were selected in this study. The face validity of the questions was ascertained by two lecturers from the Ho Technical University with expertise in environmental impact assessment. Minor textual adjustments were made without any major changes to the instrument adapted for this study. The survey questions were organized into two sections with the first part dealing with demographic information of the respondents relating to gender, age, education level, and origin of participants. The second part of the survey questions focused on the components of environmental and health effects of quarry activities. A five‐point Likert‐type scale (1 = Strongly disagree; 2 = Disagree; 3 = Neutral, 4 = Agree, and 5 = Strongly agree) was used to assess the respondent's degree of agreement with statements in the questionnaire.

## 3 Data Analysis

### 3.1 Relative Importance Index (RII)

The Relative Importance Index (RII) was used to rank the indicators in this study. The RII is one of the most reliable approaches for rating indicators using a structured questionnaire on a Likert scale (Dixit et al., 2019). The RII approach has been used in previous studies to rank exposure to hazards and environmental impacts associated with construction activities in Ghana (Boakye & Adanu, 2022; Boakye et al., 2023; Fordjour et al., 2020). The RII was calculated using the following equation:

$$\mathrm{Relative Importance Index}\;(\mathrm{RII})=\frac{\sum w}{A*N}$$

Where ⍵ is the weighting given to each factor by the respondent (ranging from 1 to 5 in this study), *A* is the highest weight (i.e., 5 in this study), and *N* is the total number of respondents (i.e., 225 in this study). The RII ranges from 0 to 1, with the highest RII indicating the maximum impact of quarrying related activities on the environment and health. The RII values have been classified into: High (H) (0.8 ≤ RI ≤ 1), High‐Medium (H‐M) (0.6 ≤ RI < 0.8), Medium (M) (0.4 ≤ RI < 0.6), Medium‐Low (M‐L) (0.2 ≤ RI < 0.4), and Low (L) (0 ≤ RI < 0.2) to determine the important levels of study indicators assessed (Akadiri, 2011; Boakye & Adanu, 2022; Boakye et al., 2023).

### 3.2 Exploratory Factor Analysis (EFA)

In order to determine the relationship among the various environmental impacts and health hazards associated with quarrying activities, an exploratory factor analysis (EFA) was used. The EFA approach is utilized to condense data into a smaller set of factors or components based on the intercorrelations of a set of variables (Pallant, 2016; Sürücü et al., 2022). The Statistical Package for the Social Sciences (SPSS version 27.0) was used to perform the EFA. The Kaiser‐Meyer‐Olkin (KMO) test was used to determine sampling adequacy. KMO values range from 0 to 1, and a minimum value of 0.5 is specified by Hair et al. (2007) as an acceptable threshold to proceed with factor analysis. Bartlett's sphericity test was used to measure the multivariate normality of the indicators and their statistical significance. We use a p‐value of <0.05 to indicate statistical significance. The key indicators in this study were chosen according to these four criteria: (a) eigenvalue 1, (b) loading values of indicators should be a minimum of 0.5, (c) one indicator should only be loaded on one factor, and (d) a factor should comprise minimum of two indicators. For detail procedure of EFA analysis refer to (Pallant, 2016; Sürücü et al., 2022).

WordItOut was used to create a word cloud visual representation of word frequency from the questionnaire responses to obtain insight into the common ailments linked to quarry activities. The frequency with which study participants brought up specific health issues is reflected in the word size.

## 4 Results

### 4.1 Demographics of Sampled Population

85.33% of the respondents were male and 14.67% were females (Table 1). The ages of respondents ranged from 18 to 65 years. One third of the respondents were 26–35 years and the least percentage age group was 18–25 years. Just over a third of the respondents completed junior high school, and only 8% had tertiary education. 81.78% of the quarry workers were natives of the study area while migrants were 18.2%.

**Table 1:** Demographic Characteristics

	Frequency	Percentage
Gender		
Male	192	85.33
Female	33	14.67
Age group of participants		
18–25	21	9.33
26–35	75	33.33
36–45	67	29.78
46–55	35	15.56
56–65	27	12.00
Education level of participants		
No formal education	31	13.78
Primary	13	5.78
Junior High School (JHS)	78	34.67
Middle School Leaving Certificate (MSLC)	26	11.56
Senior High School (SHS)	59	26.22
Tertiary	18	8.00
Origin of quarry workers		
Natives	184	81.78
Migrants	41	18.22
	225	100.00

### 4.2 Relative Importance of Exploratory Indicators

The RII of perceived impacts associated with quarrying ranged from 0.428 to 0.926 (Table 2). Water collecting in pits at quarry sites had the highest RII (0.926), followed by chest pain among quarry workers (0.916), injury from quarrying among quarry workers (0.910), loss of the beauty of the landscape as a result of quarrying (0.900), while the lowest RII scores were for skin diseases among quarry workers (0.478), and physical cracks are visible on walls of buildings as a result of quarrying (0.428). Out of the 14 indicators evaluated, six, five and three factors were of high, high to medium, and medium importance levels respectively. None of the 14 indicators evaluated fell under the low importance level.

**Table 2:** The Relative Importance Index (RII) Score, Rank and Importance Level for Quarrying Related Effects

		RII	RII rank	Importance level
1	Water collects in pits at quarry sites	0.926	1	H
2	Chest pain is common among quarry workers	0.916	2	H
3	Injury from quarrying is common among quarry workers	0.910	3	H
4	The beauty of the landscape is lost as a result of quarrying	0.900	4	H
5	The top soil is lost through quarry activities	0.891	5	H
6	The land is degraded due to quarrying	0.888	6	H
7	Eye itches and red eyes due to dust particles in the eyes of quarry workers are common	0.780	7	H‐M
8	Plants and animal species are lost due to quarrying	0.777	8	H‐M
9	Deposit of dust particles can be seen on plants close to settlements	0.670	9	H‐M
10	There are reported cases of respiratory ailments among people engaged in stone quarry activities	0.621	10	H‐M
11	Sediment from quarry sites can be seen in rivers and streams	0.602	11	H‐M
12	Cough is a common sickness among people engaged in quarrying	0.535	12	M
13	Skin diseases are common among quarry workers	0.478	13	M
14	Physical cracks are visible on walls of buildings as a result of quarrying	0.428	14	M

### 4.3 Exploratory Factor Analysis Results

On the basis of a rule of eigenvalue greater than one, and factor loadings (cut‐off at 0.5), three components were extracted after the EFA (Table 3). The 3 components extracted after the EFA accounted for 67.680% of variance explained. Components are grouping of indicators according to factor loadings. Out of the 14 indicators considered, 12 were retained based on correlation and loading on a component. Similar to Irfan et al. (2019) and Boakye et al. (2022), the indicators that did not have a significant correlation with one another were excluded from further analysis hence the removal of water collects in pits at quarry sites and plants and animal species are lost due to quarrying, respectively. The results of Cronbach's α test showed that the α value for the 12 indicators retained was 0.871, greater than 0.800, indicating a good internal consistency and reliability of the questionnaire data. Bartlett's test of sphericity score was 1250.566, and the associated significance level was *p* < 0.001 for the 12 indicators, indicating that the correlation matrix is not an identity matrix (Joseph et al., 2010).

**Table 3:** Total Variance Explained

Component	Eigenvalue	% Of variance	Cumulative %
1	5.020	41.835	41.835
2	2.097	17.471	59.306
3	1.004	8.370	67.676
4	0.693	5.775	73.451

The Kaiser‐Meyer‐Olkin (KMO) measure of all the remaining 12 indicators was 0.873, substantially greater than 0.5, and can be considered highly acceptable. Test results confirm that the sample data are appropriate for processing EFA. The total variance obtained from the factor analysis is presented in Table 3. The rotated factor loadings (cut‐off at 0.5) for the 3 factors are presented in Table 4.

**Table 4:** Summary of Exploratory Factor Analysis Results

Component	Indicators	Factor loading
1	Sediment from quarry sites can be seen in rivers and streams	0.873
	Cough is a common sickness among people engaged in quarrying	0.846
	There are reported cases of respiratory ailments among people engaged in stone quarry activities	0.844
	Skin diseases are common among quarry workers	0.795
	Deposit of dust particles can be seen on plants close to settlements	0.704
	Physical cracks are visible on walls of buildings as a result of quarrying	0.668
	Eye itches and red eyes due to dust particles in the eyes of quarry workers are common	0.643
2	The top soil is lost through quarry activities	0.852
	The land is degraded due to quarrying	0.838
	The beauty of the landscape is lost as a result of quarrying	0.689
3	Injury from quarrying is common among quarry workers	0.861
	Chest pain is common among quarry workers	0.737

Component 1 explains 41.84% of the total variance and contains seven of the 12 indicators analyzed (Table 3). The factor loadings (absolute value) on Component 1 are relatively large for sediment from quarry sites that can be seen in rivers, cough which is a common sickness among people engaged in quarrying activities (0.846) and reported cases of respiratory ailments among people engaged in stone quarry activities (Table 4).

Component 2 explains 17.47% of the total variance and comprised three indicators. The factor loadings are relatively large for the top soil is lost through quarry activities (0.852), and the land is degraded due to quarrying (0.838). Component 3 explains 8.37% of the total variance.

Component 3 comprised two indicators with the highest factor loading being injury incurred from quarrying (0.861).

### 4.4 Word Count of Common Ailments

**Figure 2:**
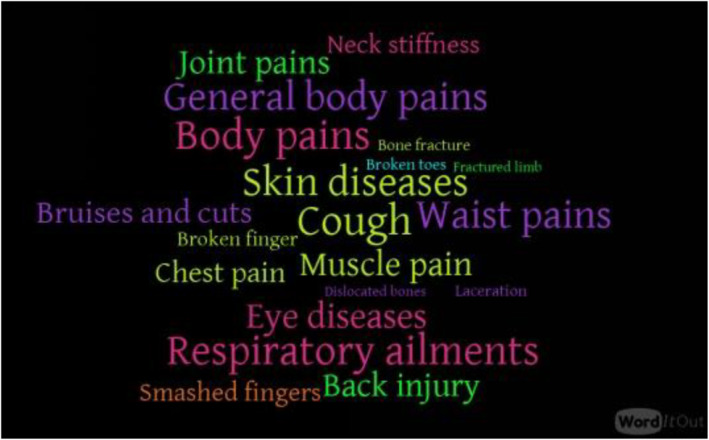
Health related issues mentioned by participants associated with quarrying activities.

Coughing (*n* = 200), was the most cited health issues associated with quarrying activities among the 20 health problems mentioned in this study. Among the most cited ailment were respiratory ailments (*n* = 195), body pains (*n* = 191), skin diseases (*n* = 190), general body pains (*n* = 182), and waist pain (*n* = 180). The least cited ailments were bone fracture (*n* = 20), fractured limb (*n* = 7), and dislocated bones (*n* = 5). Figure 2 presents a visual representation of the health issues associated with quarrying activities identified in this study. Maximum mentions are 200 for cough.

## 5 Discussions

The study on perceived effects of quarrying on the people and environment of Klefe has unearthed different perceived effects that have been ranked according to their relative perceived importance as high, high to medium and medium. The high ranking perceived effects of quarrying were collection of water in pits, chest pains, injury among quarry workers, loss of landscape beauty and top soils contributed to degradation of quarry sites. The medium to high ranked effects were eye itches, loss of plant and animal species and respiratory ailments, while the medium ranked effects were coughs, skin diseases and cracks in buildings.

The EFA results has sediments from quarry sites seen in rivers as the most ranked perceived effect responsible for damages to the environment given the highest loading factor of 0.873. Similar studies on quarry activities showed that crushing of stones produced sediments that were eroded into streams which contributed to poor water quality (Ali et al., 2020). The other high ranking effects were, coughs, skin diseases, deposit of dust particles on plants, cracks in buildings, and eye itches. In terms of the most mentioned health concerns of quarry workers, cough, body pains, skin diseases, general body pains, respiratory ailments and skin diseases were the most mentioned. Studies in other parts of the world and Ghana confirm that quarrying generates a lot of dust that causes air pollution and respiratory diseases such as bronchitis, asthma and silicosis (Ahadzi et al., 2020; Nemer et al., 2020).

The second most highly ranked perceived cause of environmental damage due to quarrying is land degradation as it explains 17.47% of the total variance. The loss of vegetation cover and the top soil has contributed to the loss of the beauty of the landscape and fertile farmlands at the quarry sites. This finding has been confirmed by a study in Kenya showing that land degradation at quarry sites caused soil erosion and gullies with average depth of 3.9 m which reduces the size of agricultural and grazing lands, causes loss of biodiversity and cut off of transport networks (Omondi et al., 2021). In the case of China, stone mining has resulted in severe environmental disturbances on the surface of rocky landscapes such as soil erosion and biodiversity decline (Qin et al., 2020).

The third most highly ranked perceived effect of quarrying in Klefe is injury from quarrying such as chest pains as it is the third highest loading factor common among quarry workers. Injuries incurred during quarrying have contributed to hospitalization of quarry workers and this keeps them away from work for days as a result affect their income. A study by Segbenya et al. (2022) shows a similar effect of poor health conditions experienced by quarry workers which has affected their well‐being and social life. In attempt to link reported aliments associated with quarrying activities to hospital data, test results of quarry workers at Wokha district in India showed their blood pressures (both systolic and diastolic), oxygen saturation (SaO_2_), pulse rate and forced vital capacity (FVC) measurements were significantly higher than measurements on people who do not engage in quarry works (Ghosh and Lohe, 2022).

## 6 Conclusion

The goal of the study was to find out the views of quarry workers and residents of Klefe regarding what they perceive as effects of quarry activities. The study showed that the majority of the quarry workers were males who were in their youthful ages and a few females. Out of the 14 factors of perceived environmental and health effects of quarrying identified, a relative importance perceived index analysis of the factors and EFA applied to the study reduced the 14 factors to three components that are considered the most important when it comes to their perceived effects on the people and their environment. These three most important perceived effects of quarrying identified were sediments from quarrying seen in streams, land degradation such as soil erosion that lead to the loss of suitable farm lands and perceived health effects. Perceived health effects of quarrying mentioned by the participant were body pains, eye itches and injuries sustained when engaging in quarrying. In attempt to address the environmental and health effects of quarrying in klefe, focus should be on sedimentation, land degradation and injuries.

## Conflict of Interest

The authors declare no conflicts of interest relevant to this study.

## Data Availability

A 4th October 2022 version of Worditout software was used. It is a free for use software owned by Enideo company of Belgium. This software is not on any repository and has no DOI number but can be accessed on this link https://worditout.com/ and it uses cookies. The trade name of the company is Kevin de Groote, with VAT number BE0502436838. A free version of SPSS 29 software was used for data analysis. This software is not kept in any repository and has no DOI number can be accessed on https://www.ibm.com/spss The developer of the software is IBM.
